# Implementation of the BETTER 2 program: a qualitative study exploring barriers and facilitators of a novel way to improve chronic disease prevention and screening in primary care

**DOI:** 10.1186/s13012-016-0525-0

**Published:** 2016-12-01

**Authors:** Nicolette Sopcak, Carolina Aguilar, Mary Ann O’Brien, Candace Nykiforuk, Kris Aubrey-Bassler, Richard Cullen, Eva Grunfeld, Donna Patricia Manca

**Affiliations:** 1 Department of Family Medicine, University of Alberta, 6-10 University Terrace, Edmonton, Alberta T6G 2T4 Canada; 2 Department of Family and Community Medicine, University of Toronto, 500 University Ave, Toronto, ON M5G 1V7 Canada; 3 School of Public Health, University of Alberta, 3-291 Edmonton Clinic Health Academy, Edmonton, AB T6G 1C9 Canada; 4 Primary Healthcare Research Unit, Memorial University of Newfoundland, Health Sciences Centre, 300 Prince Phillip Drive, St. John’s, Newfoundland, A1B 3V6 Canada; 5 Ontario Institute for Cancer Research, 661 University Avenue, Suite 510, Toronto, ON M5G 0A3 Canada; 6 Covenant Health, Grey Nuns Community Hospital, 1100 Youville Drive Northwest, Edmonton, Alberta T6L 5X8 Canada

**Keywords:** Primary care, Primary prevention, Team-based care, Health plan implementation, Qualitative research

## Abstract

**Background:**

BETTER (Building on Existing Tools to Improve Chronic Disease Prevention and Screening in Primary Care) is a patient-based intervention to improve chronic disease prevention and screening (CDPS) for cardiovascular disease, diabetes, cancer, and associated lifestyle factors in patients aged 40 to 65. The key component of BETTER is a prevention practitioner (PP), a health care professional with specialized skills in CDPS who meets with patients to develop a personalized *prevention prescription*, using the BETTER toolkit and Brief Action Planning. The purpose of this qualitative study was to understand facilitators and barriers of the implementation of the BETTER 2 program among clinicians, patients, and stakeholders in three (urban, rural, and remote) primary care settings in Newfoundland and Labrador, Canada.

**Methods:**

We collected and analyzed responses from 20 key informant interviews and 5 focus groups, as well as memos and field notes. Data were organized using Nvivo 10 software and coded using constant comparison methods. We then employed the Consolidated Framework for Implementation Research (CFIR) to focus our analysis on the domains most relevant for program implementation.

**Results:**

The following key elements, within the five CFIR domains, were identified as impacting the implementation of BETTER 2: (1) intervention characteristics—complexity and cost of the intervention; (2) outer setting—perception of fit including lack of remuneration, lack of resources, and duplication of services, as well as patients’ needs as perceived by physicians and patients; (3) characteristics of prevention practitioners—interest in prevention and ability to support and motivate patients; (4) inner setting—the availability of a local champion and working *in* a team versus working *as* a team; and (5) process—planning and engaging, collaboration, and teamwork.

**Conclusions:**

The implementation of a novel CDPS program into new primary care settings is a complex, multi-level process. This study identified key elements that hindered or facilitated the implementation of the BETTER approach in three primary care settings in Newfoundland and Labrador. Employing the CFIR as an overarching typology allows for comparisons with other contexts and settings, and may be useful for practices, researchers, and policy-makers interested in the implementation of CDPS programs.

## Background

Chronic disease prevention and screening (CDPS) has been identified as one of the top priorities in primary care [1]. Although primary care providers are commonly responsible for addressing CDPS, one study found that to address all of the recommended preventive services, an estimated 7.4 additional hours would need to be added to a physician’s regular work day [2]. BETTER (Building on Existing Tools to Improve Chronic Disease Prevention and Screening in Primary Care) is a program that aims to improve CDPS for cardiovascular disease, diabetes, cancer, and associated lifestyle factors (e.g., smoking, exercise, alcohol intake, and diet) in patients aged 40 to 65. BETTER introduces a new role, the *Prevention Practitioner* (PP), to primary care, which complements physicians’ CDPS practice. Although the role is new, the person who takes on the PP role is typically a health care provider who is already an integral part of a primary care team, such as a nurse, licensed practical nurse, or a dietician who gains specialized skills in CDPS. PPs use tools specifically developed for BETTER that are based on an extensive review and synthesis of high-level evidence for CDPS activities, which has been previously described [3, 4]. Before their prevention visit with the PP, patient participants completed the BETTER health survey, an instrument that captured a detailed prevention and screening history including risk factors such as smoking, physical activity, diet, alcohol, and family history. The PP then reviewed the patient’s medical chart and health survey to determine their individual risk for chronic disease and eligibility for screening activities. During the prevention visit, PPs developed a personalized *prevention prescription* with the patient using a collaborative process involving Brief Action Planning (BAP), an approach based on motivational interviewing and behavior change research [5]. The time commitment for the first prevention visit was approximately 90 min for both PPs and patients (for PPs: 30 min preparation + 60 min prevention visit; for patients: 30 min health survey + 60 min prevention visit). The follow-up visit was shorter and in total about 60 min for patients (30 min follow up health survey + 30 min follow-up visit) and 45 min for PPs (15 minutes preparation + 30 min follow-up visit). The BETTER trial, a pragmatic cluster randomized controlled trial, demonstrated that the prevention visits with PPs were effective in improving patients’ CDPS outcomes in the participating urban primary care settings [6]. Furthermore, a qualitative study that complemented the BETTER trial found that PPs played a key role in facilitating CDPS that benefitted both patients and primary care team members [7]. The BETTER 2 program was a second iteration based on the promising results of the BETTER trial to study the implementation of BETTER 2 as a program. Since the population in the BETTER trial reflected urban, middle class settings with a low proportion of patients with lower income or from disadvantaged settings, the purpose of the next iteration of BETTER (BETTER 2) was to pragmatically evaluate the implementation of BETTER in more diverse settings, including rural and remote, and practices caring for disadvantaged populations [8]. While our BETTER 2 sample included a higher proportion of aboriginal, rural, and remote patients (compared to the BETTER trial), overall, patients tended to be well-educated with relatively high incomes and we had only limited success in recruiting disadvantaged populations. The quantitative results of the implementation of BETTER 2, which include descriptive statistics of the clinical settings, are currently under review for publication at a different journal (Aubrey-Bassler et al., Achievement of chronic disease prevention and screening maneuvers at six-month follow-up: An implementation study of the BETTER 2 Program, manuscript submitted). The protocol for BETTER 2 [8], the knowledge synthesis and the integration process used [4] have been previously published. This paper describes the findings of the qualitative component of the program evaluation, focusing on understanding facilitators and barriers, benefits, and disadvantages of the BETTER approach in different settings in Newfoundland and Labrador, Canada.

## Methods

### Setting and participants

This qualitative study evaluated the BETTER 2 program implementation in three primary care practice settings in Newfoundland and Labrador: (1) a team practice in a remote area consisting of several physicians and other health care providers, with a licensed practical nurse (LPN) who was already part of that team taking on the PP role; (2) a rural family physician and nurse practitioner (NP) practice, in which the NP took on the PP role; and (3) an urban academic team practice in which a NP from outside of the practice joined the team as a PP (the team practice did not previously have a team member available for the role). Before implementation, PPs participated in training provided by the BETTER team, which involved an introduction to the BETTER approach and tools, the prevention visit process and Brief Action Planning. One hundred fifty-four patients participated in BETTER 2 and received personalized prevention visits with a PP. In BETTER 2, we were successful in recruiting aboriginal, rural, and remote patients, but despite including an urban practice with a high proportion of disadvantaged patients, the people enrolled from that practice tended to be well-educated and earned relatively high incomes. The several participants with lower income were recruited from the rural clinic site, however, this site experienced significant turnover in both physician and nurse practitioner coverage during the study period, which negatively affected the recruitment and retention of patients in the study. Ethics approval for BETTER 2 was received from the Health Research Ethics Board at the University of Alberta (Pro00039331) in May 2013 and the Health Research Ethics Authority of Newfoundland and Labrador (Ref # 13.120) in June 2013.

Purposeful sampling was used to ensure representation from three participant groups as part of the qualitative component of BETTER 2:

- Group 1: patients who received a personalized prevention visit with a PP received invitations to provide anonymous written feedback;
- Group 2: all primary care providers (clinicians and their staff), researchers, and administrators directly involved in the implementation of BETTER 2 were invited to key information interviews and/or focus groups through personalized invitations sent by members of the BETTER 2 team; and
- Group 3: other partners (e.g., policymakers, community partners in participating jurisdictions) indirectly involved in the implementation of BETTER 2 were invited to key informant interviews and/or focus groups through personalized invitations sent by members of the BETTER 2 team.

Data collection strategies included semi-structured interviews (one-on-one interviews and focus groups) with participants from groups 2 and 3 above by a trained researcher at three time periods throughout the study. Interviews and focus groups were conducted in person when possible, particularly in the beginning of the implementation; however, over half of the interviews were conducted by phone. While focus groups allowed for capturing different participant perspectives, key informant interviews were conducted with participants who had specific knowledge that may not have been shared in a group setting (e.g., PPs describing difficulties in their specific setting). All interviews were conducted with fully informed and written consent. Patients’ perspectives (group 1 above) were obtained using anonymous written feedback forms after each prevention visit. While we integrate some patient feedback in this paper to provide additional context to our data (i.e., how patients’ perceptions facilitated the program implementation), the data emerging from the patient feedback were collected separately and the content was not directly related to the barriers and facilitators of implementation, which is the focus of this paper. To do justice to the emerging data and to better reflect patients’ perspectives, the results based on the feedback received from patients who participated in the intervention will be discussed more extensively in a separate manuscript.

### Approach

The first round of focus groups and interviews took place shortly after implementation of the program (i.e., after PPs saw their first few patients for their first prevention visits) to assist with start-up issues and adaptations. A second round of focus groups and interviews were conducted midpoint approximately 6 months after the first round, and a third round at approximately 10 to 13 months after the first round to capture information on the feasibility and impact of BETTER 2. Participants were asked: (1) how BETTER 2 was implemented in their primary care settings, (2) their perspectives on the intervention, (3) the (anticipated and current) impact of BETTER 2, (4) barriers and enablers of BETTER 2, and (5) how the intervention could be improved. Focus groups were used to exchange and capture different perspectives in a larger group setting, while key informant interviews allowed us to obtain knowledge or perspectives that may not have emerged in a group setting.

### Analysis

Focus groups and key informant interviews were audio recorded and transcribed verbatim. Since this qualitative study used a combination of induction (bottom-up) and deduction (top-down) approach, data were coded in two phases. In the first phase of analysis, we imported and organized all data in the software program NVivo 10 [9] and coded the data using the constant comparative method informed by grounded theory [10, 11]. First, transcripts were read and coded line by line, that is, each idea was given a name or word summarizing the main idea or concept (inductive approach). Several team members with expertise in qualitative methods (NS, DM, MAO, CA) coded transcripts independently of each other and compared their codes in regular meetings to review and refine their codes and to discuss emerging themes on a higher level. Any discrepancies were resolved through team discussion. In the second phase of analysis (deductive approach), the Consolidated Framework for Implementation Research (CFIR) was employed to frame the results in a comprehensive and systematic manner by applying key domains that are considered most salient in program implementation [12]. The 5 key domains of the CFIR are as follows: (1) intervention characteristics, (2) outer setting, (3) characteristics of individuals, (4) inner setting, and (5) process. The CFIR was chosen because it is a meta-theoretical framework that was developed based on a synthesis of 200 existing frameworks and models used in health sciences [12]. Moreover, the CFIR has been specifically used to review facilitators and barriers of complex implementations in primary care [13]. In addition, use of the CFIR facilitates the comparison of implementation programs by other researchers [12].

### Rigor

Rigor was ensured by the following procedures [10, 14]: (1) consistent with the constant comparison method, team members engaged in an iterative process of data collection, data analysis, and application of the CFIR followed by further data analysis; (2) investigators used field notes and memo writing after interviews and focus groups to advance and contextualize data analysis and interpretation of findings; and (3) triangulation was achieved by collecting data from different sources (focus groups, key informant interviews, field notes, and memos) and during data analysis, as the investigators initially coded transcripts independently of each other and then met to discuss and develop consensus on codes and themes in an iterative process.

## Results

A total of 25 individuals involved in the implementation of BETTER 2 consented to participate in our qualitative study, where 5 focus groups (ranging from 3 to 9 participants each) and 20 one-on-one key informant interviews were conducted with participants. Most participants were interviewed more than once. To provide different perspectives and discussion levels between participants, focus groups varied in size and professional background of participants. For instance, some focus groups consisted only of physicians whereas others incorporated larger clinic teams. Participants included 3 PPs, 13 physicians, 4 managers, 3 researchers, 1 physiotherapist, and 1 nurse. In addition, we received 91 written feedback forms from patients who received one or more prevention visit(s). We present our findings grouped into the 5 key domains of the CFIR: (1) intervention characteristics, (2) outer setting, (3) characteristics of individuals, (4) inner setting, and (5) process. A summary of key facilitators and barriers to program implementation described below are presented in Table 1.

**Table 1:** Facilitators and Barriers of the BETTER 2 program described using the CFIR

CFIR domain	Key element	Barrier	Facilitator
Intervention characteristic	Complexity	Amount of material was perceived as overwhelming and time consuming	Strong evidence for intervention (previous RCT) Patients liked comprehensiveness (multifactorial approach as opposed to specific disease or organ)
	Cost	Perception of intervention being too costly	Perception of intervention being cost effective (investing in prevention offsets acute care costs)
Outer setting	Perception of fit	Lack of remuneration Lack of resources (particularly staff) Physicians’ perception that PP’s prevention visit is a duplication of services	Perception by other stakeholders (including managers) that CDPS is a “hot topic” Patients’ perception of visits as valuable, necessary and motivating
Characteristics of individuals	The PPs	Barrier did not emerge from data	Interest in prevention Ability to support and motivate patients
Inner setting	Local champion	Lack of local champion or losing a local champion (e.g., physician left community)	Facilitator did not emerge from data
	Working *in* a team versus working *as* a team	Not working as a team (e.g., team tensions, lack of relationship, competition, unclear roles)	Working as a team (e.g., trust; physicians appreciating PPs structuring CDPS)
Process	Planning and engaging	Not including collaborators enough in planning process	Starting collaborative conversations early
	Collaboration and teamwork	Lack of awareness/misconception of BETTER approach	Availability of team members, frequent and open conversations

### Domain 1: intervention characteristics

Based on respondents’ answers, *complexity* and *cost* emerged as important intervention characteristics that affected the implementation of BETTER 2.

### Complexity

Although many respondents liked the comprehensiveness of the intervention, its complexity was perceived as a barrier to implementation. In addition to completing forms for the visit, PPs described that they had to complete data collection forms to enable the measurement of study outcomes. Primary care providers (physicians and PPs) identified the complexity of the intervention, particularly the amount of paperwork and the time needed to collect information, as the main barriers of the intervention:

*It*’*s not something that*’*s really user friendly. And I know you need it for your data collection right now and it*’*s a part of the study* … *if I just had one page to fill out*, *document it in the EMR* [*electronic medical record*] *my life would be a lot easier*, *I would see a lot more patients*. [*PP*]

Although the complexity of the material was identified as a barrier, one facilitator in recruiting primary care teams to implement the program was its credibility. Participants commented on the program’s quality and the strength of evidence as indicated by the results of the BETTER trial [6]. For instance, one of the collaborators observed that:

*The biggest facilitator*, *I think*, *was that it was multi*-*factorial*, *just the materials that were prepared*, *the strength of the evidence from* [*the BETTER trial*], *the recognition that it was a quality program*. [*Administrator*/*Manager*]

The term complexity was interpreted differently between providers and patients. While clinicians saw the complexity as a barrier, particularly the amount of time spent on materials and paperwork, patients did not express dissatisfaction or aversion to the time commitment required on their part. Many patients interpreted the complexity and time commitment as a way of being treated comprehensively, that is from a holistic and multi-factorial approach (as opposed to being limited to a specific disease or organ). Patients’ perception was a facilitator in program implementation as they emphasized that they appreciated a health care professional taking the time to inform them about their overall health, and how the visit made them think more about taking responsibility for their health:

*There was sufficient time allotted to have a comprehensive consultation*; [*the PP*] *was excellent to talk to and listened attentively to my input. I liked the multi*-*disciplinary approach in that we discussed all* [*emphasis in original*] *aspects of my health*. [*Patient 79*]

### Cost

Cost emerged as another main theme in this domain. Although the cost of the intervention (particularly the PPs’ time) was covered by the study (funded by Canadian Partnership Against Cancer (CPAC)), participants commented on the cost as a barrier of the intervention. In particular, physicians, who typically book brief appointments, commented that having a 30–60-minute prevention appointment would be too costly.

*It*’*s just not cost effective. It*’*s gonna be outrageously expensive and not doable. That*’*s my opinion*. [*Physician*].

Interestingly, while cost was an issue mostly raised by physicians, other respondents (e.g., managers, researchers, and other health care professionals), did not see the cost as a barrier, but rather as an opportunity to save money in the long term.

[*W*]*e*’*re focused so much on acute care and really if we spent more resources on preventative care*, *we*’*d probably save more money. Devoted*, *more preventative care prevents the expense of acute care*, *right*? *And so I*’*m hopeful that patients will be more likely to adapt their lifestyle*, *lifestyle changes*, *screening tests* … *they will improve their health in the long run*. [*Researcher*]

Considering the difference in remuneration for a physician compared to a NP, a nurse, or LPN, some respondents indicated that non-physician clinicians would be better suited to providing focused CDPS visits. They also offered a perspective that extended the concept of cost beyond the actual time spent on a visit to personal cost in terms of quality of life and potential cost savings for the health care system:

*The ideal is prevention to begin with. You*’*re going to prevent a lot of secondary and tertiary need for prevention down the road*, *particularly with diabetes*, *heart disease*, *if you can prevent that from happening to begin with*, *then we won*’*t need to have clinics for people who have complications from diabetes or complications from heart disease. So if you get it from the ground up*, *then it*’*s certainly beneficial to the patients*, *but also the health care system in general* … *Not only the financial cost*, *but the human cost and the lifestyle*, *the way it affects people*’*s lifestyle*. [*Manager*]

### Domain 2: outer setting

#### Perception of fit

The perceived fit of the intervention within primary care settings varied considerably among participants. Whereas PPs, patients, and managers commonly perceived the intervention as a good fit, some physicians were more skeptical. Respondents who indicated that BETTER 2 was a good fit commented on how health care needs are universal, that CDPS is currently a *hot topic* in policy, and that the approach fits with their professional mandate (nurses, NPs). For instance, one of the managers commented on BETTER 2 as follows:

*I think that* [*BETTER 2*] *fits really. I think that it works well. I think it*’*s a good project. I think that the patients took a lot from it* … *and it made them think about their health*, *so I think it*’*s excellent*, *and I think that a patient in Newfoundland and Labrador could have the same health problems or needs as a patient in Alberta*, *so I think that it works in any setting*, *and I thought that it fit very well*. [*Manager*]

Besides primary care providers and patients subscribing to the notion that CDPS should be a focus of primary health care, administrators perceived that CDPS was a topic being discussed at a higher political level:

*I think it fits because* … *if you went to the Department of Health*, *I would guess that if you say*: ‘*What*’*s on your radar*’, *chronic disease is the thing that*’*s on their radar right now and it has been for some time. So I think* [*BETTER 2*] *came at a good time in that way*, ‘*cause that*’*s what everybody is talking about or has been for a while*. [*Manager*]

While the timing seemed to be right for the implementation of BETTER 2, the addition of a PP was also seen as a means to bridge the prevention and screening gap in primary care.

*I think your take on it was great*, *like the doctors are taking care of the acute care*, *but then we had* [*a PP*], *someone else who was trying to do a little bit of the prevention work and I think that that was probably the best way to do it in our situation*. [*Manager*]

Although many respondents saw BETTER 2 as a good fit, those who perceived BETTER 2 as not being a good fit for their setting, named three main reasons:

### Lack of remuneration

In contrast to some Canadian provinces, physicians working in Newfoundland and Labrador did not have a billing code for prevention and lifestyle counseling at the time of the study, which was identified as a barrier for implementing a CDPS program:

*I*’*m very big on prevention in my practice*, *and I think it*’*s essential* (…) *it*’*s probably the most difficult to do in Newfoundland*, *for multiple reasons. You know*, *there*’*s the time aspect*, *there*’*s also* – *where I*’*ve worked in other provinces there*’*s actually a billing code for lifestyle counseling or you*’*re allowed to do an annual physical. Umm*, *in Newfoundland you are not* … *there*’*s no recognition of the increased time* [*Physician*].

### Lack of resources

In the context of competing health care demands and scarce resources, respondents expressed that acute care trumps prevention. For instance, physicians voiced that limited resources, specifically the lack of staff, made it difficult to allocate more time or resources to CDPS as they were experiencing challenges in meeting immediate acute care needs without the aid of external resources.

*We got enough staffing problems as it is*-[*Physician 1*]//*Exactly*, *that*’*s the biggest problem is that you*’*re using the staff members* [*as PPs*] *that we have to run the clinic*. [*Physician 2*]

### Duplication of services

BETTER 2 was also perceived as not being a good fit with primary care providers who believed that they did a good job of CDPS already. Many physicians stated that CDPS was an integral part of their practice:

*I see it as an essential part of what I do* … *I spend a fair bit of time doing that with patients already*, *talking to them about diet and exercise*, *and how to approach it*, *and giving them tips* … *I do that all the time* [*Physician*].

### Patients’ needs (physicians’ perspectives)

Some physicians noted that it was difficult for a small group of eligible patients to attend an in-person prevention visit at the clinic. These patients were identified as those who would probably benefit most from a prevention visit as they were often high-risk and hard to reach.

*I guess one of the barriers* [*is that*] *some of my patients are hard to get to do preventive care*, *who don*’*t*, *you know*, *don*’*t do the mammogram. You know*, *it*’*s because they have to take the bus up to the screening centre* […] *They take a bus to come see me or they walk*; *they don*’*t drive*, *so they*’*re my patients that are less likely to come in for an hour and see a practitioner* […] *I mean there*’*s those barriers. There*’*s financial barriers for patients and time*! [*Physician*].

Although some physicians directly invited patients to book a visit with a PP, BETTER 2 was open to all eligible patients. Physicians perceived that BETTER 2 attracted patients who were already motivated to make lifestyle changes and were generally more interested in prevention and screening.

*Most of the people in my practice that don*’*t need to go*, *they are the ones that go. I think* [*the PP*] *is getting the* “*worried well*”. [*Physician*]

### Patients’ needs (patients’ perspectives)

Whereas some physicians did not see BETTER as a good fit to meet their patients’ needs, patients’ own responses were quite different. We received 91 written feedback forms from patients, who unanimously commented positively on their prevention visits. Patients appreciated that the visits were personalized as well as the time taken to go over CDPS in a comprehensive way. Patients saw prevention visits as beneficial for a variety of reasons; mainly: (1) time spent on prevention and screening—patients spent 30–60 minutes with a PP per visit, in contrast to the brief time during a typical appointment with a physician; (2) format of communication—having someone listen to them and explain tests and lab values in a comprehensive and meaningful way; and (3) empowerment—being motivated to set goals to improve their health and lifestyle. None of the patients expressed that the prevention visit with a PP was a duplication of services or that the information given at the visit was irrelevant.

*On my first visit*, *I didn*’*t have to re*-*hash a lot of stuff as* [*the PP*] *knew already. Made sure all the little issues like blood work*, *occult blood test*, *colonoscopy was done. Dr. misses these things*, *but not the* [*PP*]. *Wish I saw her on a regular basis. Top notch. Hated to finish with program*. [*Patient 37*]

Both physicians and patients identified a lack of resources and access to health care. However, while physicians perceived the PP as an addition that would take resources away from the practice in terms of workflow, patients felt that having CDPS visits with the PP helped alleviate the stress on physicians.

*Too bad the program as it was has been discontinued. I don*’*t think it is a viable option to expect GPs to carry this on when there are still people without family doctors*. [*Patient 51*]

### Domain 3: characteristics of individuals (prevention practitioners)

Data suggested that to be successful in their role, PPs need skills in time management, planning for the PP visit, and prioritizing the medical and clinical information. The PPs also needed to be good communicators and effective listeners, who are comfortable with conducting personalized one-on-one visits with patients. For this study, two NPs and one LPN were selected to take on the role of PP, based on availability, recommendations, or preference by participating clinics and/or the regional health authorities. Despite the differences in clinical training, all PPs received equally positive feedback from their supervisors and patients. Although clinical skills were necessary and part of the PP role, one of the main findings was that credentials were less important to succeeding in the PP role than the personal characteristics of the individual taking on the role. Respondents identified the following characteristics as key to success of the PP role:

### Interest in prevention

As described in the intervention characteristics, learning the approach and getting familiar with the BETTER 2 tools were perceived as complex, requiring time and motivation on the part of the PPs. All of the PPs strongly valued prevention and screening and perceived BETTER 2 to be a good fit with their clinical role and training as health professionals.

[*I*]*t definitely makes people aware*, *you know. It brings attention to the fact of prevention*, *you know*? *And that*’*s what the project is about*, *and I mean that*’*s what nursing is. That*’*s what I do*, *right*? *You know*, *so I like it*, *I really*, *really do*. [*PP*]

### Ability to support and motivate patients

Patient respondents commented on how they perceived BETTER to be different from usual care. Instead of telling patients what they should do, PPs used BAP [5] to help patients formulate S.M.A.R.T. (specific, measurable, attainable, realistic, time-based) goals [15], which puts patients in the driver’s seat to take ownership of their prevention and screening goals. Patients perceived the non-judgmental, gentle, evoking approach of the PP as supportive and motivating.

*The Prevention Practitioner listened to me*, *and she was very helpful with realistic and practical advice. She was compassionate and encouraging*. [*Patient 45*]

[*The PP was*] *friendly*, *non*-*judgmental. Assisted me with problem solving around my issues and helped me establish attainable and measurable goals within a set timeline. Enthusiastic*, *encouraging*. [*Patient 61*]

### Domain 4: inner setting

#### Local champion (or lack thereof)

Beginning with the initial stages of program implementation, local champions were identified by the BETTER 2 team as an asset:

*I think having someone on the ground*, *either at the clinic or in the community*, *depending on the setting*, *who can really be a champion for the project would be nice. You need to kind of identify that person early on and get them on board*, *otherwise there*’*s that perception of*, ‘*these people are coming in here*, *you know*, *telling us what we should be doing and we don*’*t agree*’. [*Manager*]

The importance of having a local champion to facilitate program implementation was best exemplified when one of the local champions, a physician, left a participating primary care setting and recruitment and uptake noticeably stalled as a consequence. Besides losing an advocate for the program, the physician’s leaving also had practical implications for the PP:

[*W*]*e had some challenges with turnover in doctor number 1. In order for* [*the PP*] *to practice*, *she has to have a collaborative physician* (…) *But we were running into the challenge of* [*the PP*] *not even being able to practice because our doctor left*, […] *so that was very challenging*. [*Manager*]

Moreover, having a new clinician arrive in a small community did not help with program implementation, particularly with recruiting patients for the project.

[*The PP*] *had a bit of a hard time down there because of it being such a small*, *close*-*knit community. It takes a while for* [*the patients*] *to build up their confidence in anyone*. [*Manager*]

### Working in a team versus working as a team

Being a new role to the primary care setting, the establishment of good working relationships between primary care providers and the PP was viewed as essential since unclear responsibilities/boundaries or a lack of relationship created tension and discomfort.

*If they*’*re already a team and already trust each other* … *that* [*difficult*] *dynamic wouldn*’*t exist*, *right*? *But when we brought in an outsider to the clinic*, *there was* […] *no previous trust*, *no previous relationship*. [*Administrator*/*Manager*]

[*I*]*t*’*s a great practice* (…) *but it was a little bit territorial in the beginning* (…) *Maybe I did a little bit too much in the beginning*, *and they got their back up a little tiny bit*. [*PP*]

Primary care physicians participating in BETTER 2 also engaged in CDPS activities with their patients, possibly creating an overlap with the PP role. Established trust between the PP and physician was important in negotiating this overlap. Some physicians commented on how the PP could alleviate physicians’ workload by reviewing charts and taking time to talk to the patient about lifestyle changes.

*Some doctors would say*, “*you know*, *I really appreciate the fact that somebody else is taking time to go through that because I really don*’*t have the time in the 15*-*minute appointment*”. [*PP*]

Some physicians also addressed the possible issue of competition, perceiving that the PP was taking away an important part of their role as health care professionals:

“[*T*]*he pap smear* [*visit*] *is my one sole time I feel good about the care I deliver because I try to cover all my bases at that visit*, *right*, *so that is my check and the whole health promotion piece*, *and the whole population health and prevention piece is big and important to me*” [*Physician*].

### Domain 5: process

Although many important activities are involved in the process of program implementation, two activities emerged as essential for the implementation of BETTER 2: planning and engaging, and interprofessional collaboration. While grounded in data, in this section we reflect at a more conceptual level on our learnings during the implementation process.

### Planning and engaging

In an effort to allocate time and resources wisely, a concise presentation of the BETTER approach outlining the BETTER trial results, and the purpose and planned implementation process for BETTER 2 were used when engaging primary care settings at the beginning of the program. Upon reflection, the BETTER 2 team recognized that while clear aims were important, providing an opportunity to adapt and modify some elements of the approach may have been equally important. Starting collaborative conversations early may increase stakeholders’ (particularly primary care providers’ and managers’) desire to join a project that they can adapt to their needs:

*When I first approached them about BETTER 2*, *the way I went about it was to*, *you know*, *basically spoon*-*fed to them because I figured that any work on their part might be a barrier*, ‘*cause they*’*re just overextended as it is. They have too much on their plate*, *and I have no way of knowing of course*, *what would have happened*, *but if I had used a little bit different approach*, *left things open and really tried to engage them in redesigning BETTER to suit their needs then that may have worked out better in the long run. They might have had more investment in the process and therefore*, *more investment in sustaining it as well*. [*Researcher*]

Early engagement of administrators and managers alongside primary care providers in the first round of interviews and focus groups helped to integrate the activities and ensure that everyone was on the same page. For the implementation, we learned that clarifying what the PP role entailed (e.g., scope, what guidelines they used) and to hear expectations from primary care providers would have helped to increase engagement and optimize communication within primary care teams.

### Collaboration and teamwork

The BETTER 2 program implementation involved stakeholders from different disciplines including administrators, researchers, nurses, physicians, and managers. Since each discipline comes with its own practices, team culture and agenda, collaboration and teamwork need to be examined and negotiated. Furthermore, introducing a new approach impacts the routine and the workflow of a primary care setting, and it requires people to do things in a new way or incorporate a new component into their practice. Frequent conversations are necessary to identify issues and keep implementation on track.

*After a lot of phone calls and a lot of e*-*mails and juggling of the expectations*, *it seems like we were able to figure out something that works for everybody*. [*Coordinator*]

## Discussion

The unique characteristic of BETTER is the comprehensive approach used to address a wide variety of maneuvers related to a patient’s health, such as blood results, diet, physical activity, mental health, in a one-hour prevention visit with a PP [6, 7]. While this qualitative study suggests that the implementation of BETTER 2 was valued as an opportunity to address patients’ complex and multi-faceted health care needs from a CDPS perspective, it also revealed important barriers related to implementing comprehensive preventive activities. Our results identified a disconnect between perceptions of physicians regarding the BETTER 2 program and those of other primary care providers and patients. This reflects the long-standing recognition that physicians and patients often have different positions and perspectives of illness and care [16]. For instance, patients appreciated the individualized prevention visits with PPs and described them as motivating, non-judgmental, and supportive. Some physicians, on the other hand, emphasized pride in being able to provide CDPS as part of their regular practice, and expressed a preference to provide those services themselves.

While BETTER 2 conceptualizes the role of the PP as complementary to physicians’ CDPS practice, some physicians felt that the addition of a PP took away a part of their work that they enjoyed, which created tension in some teams [17]. The resistance of primary care providers to implement complex interventions in the context of prevention has been identified in the literature [18]. One important reason for this barrier may be the disconnect on a systemic level between the pursued ideal of patient-centered comprehensive preventive care and the reality of a health care system that currently focuses on episodic and acute care [13]. Rubio-Valera and colleagues concluded in their synthesis review of qualitative research on primary prevention and health promotion that: “Primary care is perceived as well-placed to implement primary prevention and health promotion but workload, lack of time and referral resources, and the predominance of the biomedical model (which prioritizes disease treatment) hamper the implementation of primary prevention and health promotion” (2004, p. 1) [18]. Although preventive care is known to lower the need for acute care, and therefore health care cost, adequate time for prevention and screening is often unavailable due to patients’ acute care demands and scarcity of health care resources in some communities [19]. BETTER 2 provided the opportunity to overcome this barrier by introducing a PP to the primary care setting to facilitate the integration of CDPS in a structured and streamlined way, freeing up physicians’ time to focus on patients’ acute care needs. However, our findings suggest that this approach may work best when roles are clearly negotiated and primary care providers work well together as a team. Finally, upon reflecting on the process of the BETTER 2 implementation, we found that while collaboration and teamwork may be time consuming, it is necessary to set aside time to discuss expectations and ongoing processes with stakeholders in order to foster positive collaboration and teamwork in diverse primary care settings [20]. We chose the CFIR, as it is a meta-theoretical framework that was developed based on a synthesis of 200 existing frameworks and models used in health sciences [12]. Employing the CFIR was valuable to organize our evaluation and helped describe key facilitators and barriers to implementation of the BETTER 2 program. However, although the CFIR helped us to conceptualize key elements at a higher level, it had its limitations as well as it fell short in allowing us to describe the specifics of the different primary contexts (e.g., urban, rural, and remote).

### Strength

This qualitative study explored the perspectives of a diverse group of stakeholders (primary care providers, administrators, managers, and patients) from diverse settings (urban, rural, remote) involved in the implementation of the BETTER 2 program. Respondents’ perspectives provided valuable insights into the contextual factors that influenced implementation as well as differing views and perceptions regarding the benefit to and need of a CDPS program. Collecting data at three different points in time—at the beginning, mid-way, and at the end of program implementation—allowed for integration of different perspectives and identification of key factors that needed to be addressed in order to facilitate the implementation of a CDPS program.

### Limitations

Participation in the qualitative evaluation the BETTER 2 program was voluntary and limited to stakeholders who had been involved in the implementation of the program in Newfoundland and Labrador. It is possible that the perspectives of the individuals who participated in the qualitative evaluation of BETTER 2 may differ from those of stakeholders who did not participate in the program implementation. Furthermore, our sample consisted of three primary care settings in Newfoundland and Labrador. Although the selected clinic sites were diverse (i.e., urban, rural, and remote), our results and representations are limited to those specific contexts and may not be representative of other primary care settings.

## Conclusion

The qualitative results described in this paper are an important contribution to our understanding of key factors necessary for a successful implementation of the BETTER 2 program. While this study also confirmed what we learned from the qualitative study associated with the BETTER trial, namely, that introducing the new role of a PP changed dynamics in primary care settings for patients, the primary care team, and other primary care providers [7], it also explored contextual nuances and shed light on different perspectives related to the implementation in a different primary care setting. Early engagement facilitated program uptake and improved the ability to better adapt the program to the needs of the participants. With a growing demand for increased integration of CDPS in primary care, we believe that our insights around implementation of the BETTER 2 program will inform and may benefit other primary care teams who consider or are in the process of integrating CDPS approaches into their practice.

## Abbreviations

BAP: Brief action planning
BETTER 2: Building on existing tools to improve chronic disease prevention and screening in primary care (implementation study)
BETTER trial: Building on existing tools to improve chronic disease prevention and screening in primary care (a pragmatic cluster randomized controlled trial)
CDPS: Chronic disease prevention and screening
CWG: Clinical working group
EMR: Electronic medical record
LPN: Licensed practical nurse
MI: Motivational interviewing
NP: Nurse practitioner
PP: Prevention practitioner
S.M.A.R.T: Specific, measurable, attainable, realistic, time-based
